# 4-Chloro­benzaldehyde (1-isobutyl-1*H*-imidazo[4,5-*c*]quinolin-4-yl)hydrazone monohydrate

**DOI:** 10.1107/S1600536811001577

**Published:** 2011-01-15

**Authors:** Wan-Sin Loh, Hoong-Kun Fun, Reshma Kayarmar, S. Viveka, G. K. Nagaraja

**Affiliations:** a X-ray Crystallography Unit, School of Physics, Universiti Sains Malaysia, 11800 USM, Penang, Malaysia; b Department of Chemistry, Mangalore University, Karnataka, India

## Abstract

In the title compound, C_21_H_20_ClN_5_·H_2_O, the 1*H*-imidazo[4,5-*c*]quinoline ring is approximately planar, with a maximum deviation of 0.0795 (7) Å, and it forms a dihedral angle of 7.65 (3)° with the chloro­phenyl ring. In the crystal, the components are linked into chains along the *a* axis *via* inter­molecular N—H⋯O, O—H⋯N and C—H⋯O hydrogen bonds. One of the H atoms of the water mol­ecule is disordered over two positions with a site-occupancy ratio of 0.80 (4):0.20 (4).

## Related literature

For background to quinolines and their microbial activity, see: El-Subbagh *et al.* (2000 ▶); Atwell *et al.* (1989 ▶); Kuo *et al.* (1993 ▶); Xia *et al.* (1998 ▶). For the biological activity of Schiff base hydrazones, see: Colins & Lyne (1970 ▶); Ochiai (1977 ▶). For bond-length data, see: Allen *et al.* (1987 ▶). For related structures, see: Loh *et al.* (2011**a* ▶,b* ▶). For the stability of the temperature controller used in the data collection, see: Cosier & Glazer (1986 ▶).
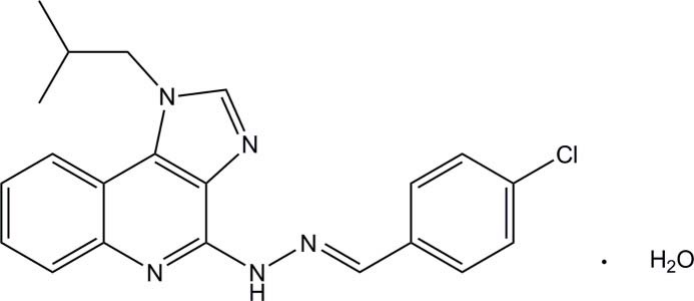


## Experimental

### 

#### Crystal data

C_21_H_20_ClN_5_·H_2_O
*M* *_r_* = 395.89
Monoclinic, 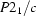

*a* = 10.4117 (3) Å
*b* = 18.2365 (6) Å
*c* = 11.9019 (3) Å
β = 117.809 (2)°
*V* = 1998.85 (10) Å^3^
*Z* = 4
Mo *K*α radiation
μ = 0.21 mm^−1^
*T* = 100 K
0.49 × 0.45 × 0.18 mm

#### Data collection

Bruker SMART APEXII DUO CCD area-detector diffractometer
Absorption correction: multi-scan (*SADABS*; Bruker, 2009 ▶) *T*_min_ = 0.904, *T*_max_ = 0.963
39468 measured reflections
10411 independent reflections
8351 reflections with *I* > 2σ(*I*)
*R*_int_ = 0.032

#### Refinement

*R*[*F* ^2^ > 2σ(*F* ^2^)] = 0.045
*wR*(*F* ^2^) = 0.135
*S* = 1.04
10411 reflections
260 parameters
H atoms treated by a mixture of independent and constrained refinement
Δρ_max_ = 1.19 e Å^−3^
Δρ_min_ = −0.47 e Å^−3^

### 

Data collection: *APEX2* (Bruker, 2009 ▶); cell refinement: *SAINT* (Bruker, 2009 ▶); data reduction: *SAINT*; program(s) used to solve structure: *SHELXTL* (Sheldrick, 2008 ▶); program(s) used to refine structure: *SHELXTL*; molecular graphics: *SHELXTL*; software used to prepare material for publication: *SHELXTL* and *PLATON* (Spek, 2009 ▶).

## Supplementary Material

Crystal structure: contains datablocks global, I. DOI: 10.1107/S1600536811001577/is2658sup1.cif

Structure factors: contains datablocks I. DOI: 10.1107/S1600536811001577/is2658Isup2.hkl

Additional supplementary materials:  crystallographic information; 3D view; checkCIF report

## Figures and Tables

**Table 1:** Hydrogen-bond geometry (Å, °)

*D*—H⋯*A*	*D*—H	H⋯*A*	*D*⋯*A*	*D*—H⋯*A*
N4—H1*N*4⋯O1*W*^i^	0.874 (19)	2.559 (18)	3.2789 (13)	140.2 (14)
O1*W*—H1*W*1⋯N1^ii^	0.83	2.09	2.9178 (14)	173
C10—H10*A*⋯O1*W*^iii^	0.93	2.52	3.3513 (16)	149
C18—H18*B*⋯O1*W*^iii^	0.97	2.59	3.4776 (14)	153

Symmetry codes: (i) 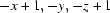; (ii) 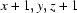; (iii) 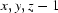.

**Fig. 1.**
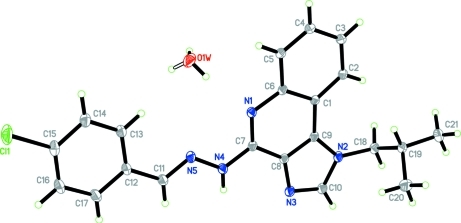
The molecular structure of the title compound, showing 50% probability displacement ellipsoids and the atom-numbering scheme. The open bond indicates the minor component.

**Fig. 2.**
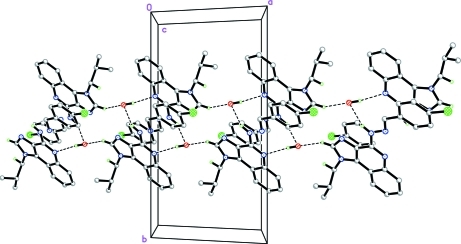
The crystal packing of the title compound, showing the chains along the a axis. H atoms not involved in the intermolecular interactions (dashed lines) have been omitted for clarity. Only major component is shown.

## supplementary crystallographic information

### Comment

Quinolines and their derivatives are important constituents of pharmacologically active synthetic compounds as these systems have been associated with a wide spectrum of biological properties (El-Subbagh *et al.*, 2000) such as DNA binding capability (Atwell *et al.*, 1989) and antitumor activities (Kuo *et al.*, 1993; Xia *et al.*, 1998). The study of Schiff base hydrazones has been growing because of their antimicrobial, anti-tuberculosis and anti-tumour activities (Colins & Lyne, 1970; Ochiai, 1977).

The asymmetric unit of the title compound, (Fig. 1), consists of one 4-chlorobenzaldehyde(1-isobutyl-1*H*-imidazo[4,5-*c*]quinolin-4-yl) hydrazone molecule and one water molecule. One of the H atoms attached to the water molecule is disordered over two positions with the site occupancy ratio of 0.80 (4):0.20 (4). The 1*H*-imidazo[4,5-*c*]quinoline ring (C1–C6/N1/C7/C8/N3/C10/N2/C9) is approximately planar with a maximum deviation of 0.0795 (7) Å at atom C2 and it forms a dihedral angle of 7.65 (3)° with the chlorophenyl ring (Cl1/C11–C16) with maximum deviation of 0.0286 (3) Å at atom Cl1. Bond lengths (Allen *et al.*, 1987) and angles are within the normal ranges and are comparable to the related structures (Loh *et al.*, 2011**a*,b*).

In the crystal packing (Fig. 2), the molecules are linked into chains along the *a* axis by the water molecules *via* intermolecular N4—H1N4···O1W, O1W—H1W1···N1, C10—H10A···O1W and C18—H18B···O1W hydrogen bonds (Table 1).

### Experimental

A mixture of 4-hydrazino-1-isobutyl-1*H*-imidazo[4,5-*c*]quinoline (2.5 g, 0.0098 mole) and 4-chlorobenzaldehyde (1.38 g, 0.0098 mole) in absolute ethanol was refluxed for 4 h in the presence of acetic acid (1 ml). The product, 4-chlorobenzaldehyde (1-isobutyl-1*H*-imidazo[4,5-*c*]quinolin-4-yl)hydrazone, was obtained after cooling and it was crystallized from absolute ethanol. Yield: 3.4 g (80%). Crystals suitable for X-ray analysis were obtained from ethanol by slow evaporation.

### Refinement

O- and N-bound H atoms were located from a difference Fourier map. O-bound H atoms were then fixed at their found positions (O—H = 0.8330 to 0.8554 Å), with *U*_iso_(H) = 1.5*U*_eq_(O), whereas N-bound H atoms was refined freely [N—H = 0.875 (18) Å]. The remaining H atoms were positioned geometrically with the bond lengths of C—H = 0.93 to 0.98 Å and were refined using a riding model, with *U*_iso_(H) = 1.2 or 1.5*U*_eq_(C). A rotating group model was applied to the methyl groups. The heighest residual electron density peak is located 1.01 Å from atom O1W.

### Crystal data

**Table:**

C_21_H_20_ClN_5_·H_2_O	*F*(000) = 832
*M**_r_* = 395.89	*D*_x_ = 1.316 Mg m^−^^3^
Monoclinic, *P*2_1_/*c*	Mo *K*α radiation, λ = 0.71073 Å
Hall symbol: -P 2ybc	Cell parameters from 9978 reflections
*a* = 10.4117 (3) Å	θ = 4.0–37.5°
*b* = 18.2365 (6) Å	µ = 0.21 mm^−^^1^
*c* = 11.9019 (3) Å	*T* = 100 K
β = 117.809 (2)°	Plate, yellow
*V* = 1998.85 (10) Å^3^	0.49 × 0.45 × 0.18 mm
*Z* = 4	

### Data collection

**Table:**

Bruker SMART APEXII DUO CCD area-detector diffractometer	10411 independent reflections
Radiation source: fine-focus sealed tube	8351 reflections with *I* > 2σ(*I*)
graphite	*R*_int_ = 0.032
φ and ω scans	θ_max_ = 37.6°, θ_min_ = 4.0°
Absorption correction: multi-scan (*SADABS*; Bruker, 2009)	*h* = −17→17
*T*_min_ = 0.904, *T*_max_ = 0.963	*k* = −31→30
39468 measured reflections	*l* = −20→20

### Refinement

**Table:**

Refinement on *F*^2^	Primary atom site location: structure-invariant direct methods
Least-squares matrix: full	Secondary atom site location: difference Fourier map
*R*[*F*^2^ > 2σ(*F*^2^)] = 0.045	Hydrogen site location: inferred from neighbouring sites
*wR*(*F*^2^) = 0.135	H atoms treated by a mixture of independent and constrained refinement
*S* = 1.04	*w* = 1/[σ^2^(*F*_o_^2^) + (0.0715*P*)^2^ + 0.4845*P*] where *P* = (*F*_o_^2^ + 2*F*_c_^2^)/3
10411 reflections	(Δ/σ)_max_ = 0.001
260 parameters	Δρ_max_ = 1.19 e Å^−^^3^
0 restraints	Δρ_min_ = −0.47 e Å^−^^3^

### Special details

**Table:**

Experimental. The crystal was placed in the cold stream of an Oxford Cryosystems Cobra open-flow nitrogen cryostat (Cosier & Glazer, 1986) operating at 100.0 (1) K.
Geometry. All e.s.d.'s (except the e.s.d. in the dihedral angle between two l.s. planes) are estimated using the full covariance matrix. The cell e.s.d.'s are taken into account individually in the estimation of e.s.d.'s in distances, angles and torsion angles; correlations between e.s.d.'s in cell parameters are only used when they are defined by crystal symmetry. An approximate (isotropic) treatment of cell e.s.d.'s is used for estimating e.s.d.'s involving l.s. planes.
Refinement. Refinement of *F*^2^ against ALL reflections. The weighted *R*-factor *wR* and goodness of fit *S* are based on *F*^2^, conventional *R*-factors *R* are based on *F*, with *F* set to zero for negative *F*^2^. The threshold expression of *F*^2^ > σ(*F*^2^) is used only for calculating *R*-factors(gt) *etc*. and is not relevant to the choice of reflections for refinement. *R*-factors based on *F*^2^ are statistically about twice as large as those based on *F*, and *R*- factors based on ALL data will be even larger.

### Fractional atomic coordinates and isotropic or equivalent isotropic displacement parameters (Å^2^)

**Table:**

	*x*	*y*	*z*	*U*_iso_*/*U*_eq_	Occ. (<1)
Cl1	−0.37283 (3)	−0.053039 (16)	0.46655 (3)	0.03357 (7)	
N1	0.00417 (7)	0.13498 (4)	0.01185 (6)	0.01373 (11)	
N2	0.33923 (7)	0.14996 (4)	−0.08204 (7)	0.01514 (11)	
N3	0.35384 (7)	0.06015 (4)	0.05254 (7)	0.01630 (12)	
N4	0.13161 (8)	0.03567 (4)	0.13737 (7)	0.01564 (11)	
N5	0.02778 (7)	0.02713 (4)	0.17545 (6)	0.01482 (11)	
C1	0.09304 (8)	0.20371 (4)	−0.11917 (7)	0.01242 (11)	
C2	0.06369 (8)	0.25958 (4)	−0.21028 (7)	0.01464 (12)	
H2A	0.1260	0.2664	−0.2455	0.018*	
C3	−0.05646 (8)	0.30406 (4)	−0.24743 (7)	0.01589 (12)	
H3A	−0.0749	0.3407	−0.3075	0.019*	
C4	−0.15108 (8)	0.29408 (4)	−0.19448 (8)	0.01637 (13)	
H4A	−0.2297	0.3255	−0.2171	0.020*	
C5	−0.12834 (8)	0.23813 (4)	−0.10935 (8)	0.01560 (12)	
H5A	−0.1932	0.2314	−0.0768	0.019*	
C6	−0.00740 (8)	0.19089 (4)	−0.07104 (7)	0.01287 (11)	
C7	0.11700 (8)	0.09093 (4)	0.05390 (7)	0.01293 (11)	
C8	0.22740 (8)	0.09992 (4)	0.01609 (7)	0.01314 (11)	
C9	0.21572 (8)	0.15557 (4)	−0.06761 (7)	0.01280 (11)	
C10	0.41677 (9)	0.09246 (4)	−0.00837 (8)	0.01736 (13)	
H10A	0.5054	0.0774	−0.0017	0.021*	
C11	0.04705 (9)	−0.02385 (4)	0.25628 (7)	0.01614 (13)	
H11A	0.1279	−0.0543	0.2850	0.019*	
C12	−0.05978 (9)	−0.03357 (4)	0.30283 (7)	0.01541 (12)	
C13	−0.18721 (9)	0.00855 (4)	0.25432 (7)	0.01694 (13)	
H13A	−0.2067	0.0415	0.1887	0.020*	
C14	−0.28457 (10)	0.00168 (5)	0.30303 (8)	0.01947 (14)	
H14A	−0.3690	0.0296	0.2704	0.023*	
C15	−0.25383 (10)	−0.04775 (5)	0.40152 (8)	0.02080 (15)	
C16	−0.13059 (11)	−0.09132 (5)	0.44958 (8)	0.02182 (15)	
H16A	−0.1126	−0.1248	0.5142	0.026*	
C17	−0.03391 (10)	−0.08415 (5)	0.39943 (8)	0.01941 (14)	
H17A	0.0488	−0.1134	0.4306	0.023*	
C18	0.38977 (9)	0.19795 (4)	−0.15243 (8)	0.01655 (13)	
H18A	0.3124	0.2040	−0.2385	0.020*	
H18B	0.4711	0.1749	−0.1570	0.020*	
C19	0.43656 (8)	0.27358 (4)	−0.09030 (8)	0.01657 (13)	
H19A	0.3546	0.2959	−0.0839	0.020*	
C20	0.56378 (11)	0.26719 (6)	0.04269 (9)	0.02498 (17)	
H20A	0.5932	0.3153	0.0784	0.037*	
H20B	0.6435	0.2433	0.0383	0.037*	
H20C	0.5349	0.2390	0.0952	0.037*	
C21	0.47505 (11)	0.32187 (6)	−0.17470 (10)	0.02624 (18)	
H21A	0.5008	0.3700	−0.1382	0.039*	
H21B	0.3929	0.3253	−0.2576	0.039*	
H21C	0.5556	0.3008	−0.1815	0.039*	
H1N4	0.2039 (19)	0.0049 (9)	0.1601 (16)	0.035 (4)*	
O1W	0.70320 (9)	0.09494 (4)	0.93489 (12)	0.0421 (3)	
H1W1	0.7912	0.1025	0.9595	0.063*	
H2WA	0.6633	0.0764	0.8603	0.063*	0.80 (4)
H2WB	0.6915	0.0492	0.9405	0.063*	0.20 (4)

### Atomic displacement parameters (Å^2^)

**Table:**

	*U*^11^	*U*^22^	*U*^33^	*U*^12^	*U*^13^	*U*^23^
Cl1	0.03921 (14)	0.03795 (14)	0.03987 (14)	−0.00160 (10)	0.03211 (12)	0.00615 (10)
N1	0.0138 (2)	0.0145 (2)	0.0160 (2)	0.00128 (19)	0.0096 (2)	0.0020 (2)
N2	0.0138 (2)	0.0149 (3)	0.0216 (3)	0.0012 (2)	0.0124 (2)	0.0016 (2)
N3	0.0137 (3)	0.0151 (3)	0.0227 (3)	0.0020 (2)	0.0107 (2)	0.0021 (2)
N4	0.0154 (3)	0.0166 (3)	0.0188 (3)	0.0029 (2)	0.0112 (2)	0.0044 (2)
N5	0.0165 (3)	0.0153 (3)	0.0161 (3)	−0.0005 (2)	0.0104 (2)	0.0009 (2)
C1	0.0114 (3)	0.0139 (3)	0.0136 (3)	0.0000 (2)	0.0072 (2)	0.0001 (2)
C2	0.0139 (3)	0.0168 (3)	0.0152 (3)	0.0000 (2)	0.0085 (2)	0.0017 (2)
C3	0.0142 (3)	0.0177 (3)	0.0162 (3)	0.0010 (2)	0.0074 (2)	0.0034 (2)
C4	0.0134 (3)	0.0168 (3)	0.0196 (3)	0.0019 (2)	0.0083 (2)	0.0032 (2)
C5	0.0134 (3)	0.0169 (3)	0.0197 (3)	0.0020 (2)	0.0103 (2)	0.0029 (2)
C6	0.0122 (3)	0.0142 (3)	0.0147 (3)	0.0002 (2)	0.0084 (2)	0.0004 (2)
C7	0.0132 (3)	0.0136 (3)	0.0141 (3)	−0.0003 (2)	0.0081 (2)	−0.0001 (2)
C8	0.0121 (3)	0.0132 (3)	0.0163 (3)	0.0004 (2)	0.0084 (2)	0.0003 (2)
C9	0.0120 (3)	0.0138 (3)	0.0154 (3)	−0.0003 (2)	0.0087 (2)	−0.0003 (2)
C10	0.0149 (3)	0.0157 (3)	0.0258 (3)	0.0025 (2)	0.0132 (3)	0.0025 (3)
C11	0.0175 (3)	0.0161 (3)	0.0162 (3)	0.0004 (2)	0.0090 (2)	0.0025 (2)
C12	0.0187 (3)	0.0150 (3)	0.0142 (3)	−0.0020 (2)	0.0091 (2)	0.0007 (2)
C13	0.0193 (3)	0.0173 (3)	0.0167 (3)	−0.0005 (2)	0.0105 (3)	0.0020 (2)
C14	0.0211 (3)	0.0198 (3)	0.0218 (3)	−0.0012 (3)	0.0136 (3)	0.0011 (3)
C15	0.0261 (4)	0.0216 (3)	0.0209 (3)	−0.0049 (3)	0.0162 (3)	−0.0001 (3)
C16	0.0277 (4)	0.0224 (4)	0.0189 (3)	−0.0022 (3)	0.0138 (3)	0.0045 (3)
C17	0.0228 (4)	0.0192 (3)	0.0175 (3)	0.0001 (3)	0.0104 (3)	0.0043 (3)
C18	0.0162 (3)	0.0188 (3)	0.0200 (3)	0.0003 (2)	0.0130 (3)	0.0017 (2)
C19	0.0139 (3)	0.0177 (3)	0.0198 (3)	0.0002 (2)	0.0094 (3)	0.0031 (2)
C20	0.0216 (4)	0.0287 (4)	0.0210 (4)	−0.0013 (3)	0.0070 (3)	0.0022 (3)
C21	0.0255 (4)	0.0260 (4)	0.0297 (4)	−0.0023 (3)	0.0150 (4)	0.0094 (3)
O1W	0.0217 (3)	0.0222 (3)	0.0865 (8)	0.0064 (3)	0.0287 (4)	0.0188 (4)

### Geometric parameters (Å, °)

**Table:**

Cl1—C15	1.7430 (9)	C11—H11A	0.9300
N1—C7	1.3143 (10)	C12—C17	1.3985 (11)
N1—C6	1.3842 (9)	C12—C13	1.4032 (12)
N2—C10	1.3638 (10)	C13—C14	1.3884 (11)
N2—C9	1.3782 (9)	C13—H13A	0.9300
N2—C18	1.4684 (10)	C14—C15	1.3924 (12)
N3—C10	1.3210 (10)	C14—H14A	0.9300
N3—C8	1.3836 (10)	C15—C16	1.3857 (14)
N4—N5	1.3622 (9)	C16—C17	1.3955 (12)
N4—C7	1.3728 (10)	C16—H16A	0.9300
N4—H1N4	0.875 (18)	C17—H17A	0.9300
N5—C11	1.2845 (10)	C18—C19	1.5330 (12)
C1—C2	1.4135 (10)	C18—H18A	0.9700
C1—C6	1.4267 (10)	C18—H18B	0.9700
C1—C9	1.4308 (10)	C19—C20	1.5222 (12)
C2—C3	1.3793 (11)	C19—C21	1.5233 (12)
C2—H2A	0.9300	C19—H19A	0.9800
C3—C4	1.4072 (11)	C20—H20A	0.9600
C3—H3A	0.9300	C20—H20B	0.9600
C4—C5	1.3783 (11)	C20—H20C	0.9600
C4—H4A	0.9300	C21—H21A	0.9600
C5—C6	1.4142 (10)	C21—H21B	0.9600
C5—H5A	0.9300	C21—H21C	0.9600
C7—C8	1.4259 (10)	O1W—H1W1	0.8330
C8—C9	1.3872 (10)	O1W—H2WA	0.8554
C10—H10A	0.9300	O1W—H2WB	0.8508
C11—C12	1.4665 (11)		
			
C7—N1—C6	119.13 (6)	C17—C12—C11	120.07 (7)
C10—N2—C9	106.49 (6)	C13—C12—C11	121.10 (7)
C10—N2—C18	124.26 (6)	C14—C13—C12	120.87 (7)
C9—N2—C18	129.08 (6)	C14—C13—H13A	119.6
C10—N3—C8	103.72 (6)	C12—C13—H13A	119.6
N5—N4—C7	119.18 (6)	C13—C14—C15	118.96 (8)
N5—N4—H1N4	121.7 (12)	C13—C14—H14A	120.5
C7—N4—H1N4	119.0 (12)	C15—C14—H14A	120.5
C11—N5—N4	117.46 (7)	C16—C15—C14	121.57 (8)
C2—C1—C6	119.31 (6)	C16—C15—Cl1	119.88 (6)
C2—C1—C9	126.97 (6)	C14—C15—Cl1	118.54 (7)
C6—C1—C9	113.71 (6)	C15—C16—C17	118.91 (8)
C3—C2—C1	120.60 (7)	C15—C16—H16A	120.5
C3—C2—H2A	119.7	C17—C16—H16A	120.5
C1—C2—H2A	119.7	C16—C17—C12	120.84 (8)
C2—C3—C4	120.01 (7)	C16—C17—H17A	119.6
C2—C3—H3A	120.0	C12—C17—H17A	119.6
C4—C3—H3A	120.0	N2—C18—C19	112.26 (6)
C5—C4—C3	120.59 (7)	N2—C18—H18A	109.2
C5—C4—H4A	119.7	C19—C18—H18A	109.2
C3—C4—H4A	119.7	N2—C18—H18B	109.2
C4—C5—C6	120.65 (7)	C19—C18—H18B	109.2
C4—C5—H5A	119.7	H18A—C18—H18B	107.9
C6—C5—H5A	119.7	C20—C19—C21	111.16 (7)
N1—C6—C5	116.55 (6)	C20—C19—C18	111.05 (7)
N1—C6—C1	124.78 (6)	C21—C19—C18	108.91 (7)
C5—C6—C1	118.67 (6)	C20—C19—H19A	108.5
N1—C7—N4	120.12 (6)	C21—C19—H19A	108.5
N1—C7—C8	121.23 (7)	C18—C19—H19A	108.5
N4—C7—C8	118.64 (6)	C19—C20—H20A	109.5
N3—C8—C9	111.13 (6)	C19—C20—H20B	109.5
N3—C8—C7	129.05 (7)	H20A—C20—H20B	109.5
C9—C8—C7	119.81 (6)	C19—C20—H20C	109.5
N2—C9—C8	105.12 (6)	H20A—C20—H20C	109.5
N2—C9—C1	133.65 (7)	H20B—C20—H20C	109.5
C8—C9—C1	121.23 (6)	C19—C21—H21A	109.5
N3—C10—N2	113.54 (7)	C19—C21—H21B	109.5
N3—C10—H10A	123.2	H21A—C21—H21B	109.5
N2—C10—H10A	123.2	C19—C21—H21C	109.5
N5—C11—C12	119.30 (7)	H21A—C21—H21C	109.5
N5—C11—H11A	120.3	H21B—C21—H21C	109.5
C12—C11—H11A	120.3	H1W1—O1W—H2WA	110.7
C17—C12—C13	118.81 (7)	H1W1—O1W—H2WB	107.9
			
C7—N4—N5—C11	−178.12 (7)	N3—C8—C9—N2	−0.42 (9)
C6—C1—C2—C3	3.55 (11)	C7—C8—C9—N2	178.87 (7)
C9—C1—C2—C3	−177.20 (7)	N3—C8—C9—C1	179.51 (7)
C1—C2—C3—C4	0.05 (12)	C7—C8—C9—C1	−1.21 (11)
C2—C3—C4—C5	−2.71 (12)	C2—C1—C9—N2	3.78 (14)
C3—C4—C5—C6	1.68 (12)	C6—C1—C9—N2	−176.94 (8)
C7—N1—C6—C5	−177.76 (7)	C2—C1—C9—C8	−176.12 (7)
C7—N1—C6—C1	2.14 (11)	C6—C1—C9—C8	3.17 (10)
C4—C5—C6—N1	−178.17 (7)	C8—N3—C10—N2	−0.36 (9)
C4—C5—C6—C1	1.94 (11)	C9—N2—C10—N3	0.12 (10)
C2—C1—C6—N1	175.61 (7)	C18—N2—C10—N3	175.72 (7)
C9—C1—C6—N1	−3.74 (10)	N4—N5—C11—C12	178.17 (7)
C2—C1—C6—C5	−4.50 (11)	N5—C11—C12—C17	−174.05 (8)
C9—C1—C6—C5	176.15 (7)	N5—C11—C12—C13	4.30 (12)
C6—N1—C7—N4	179.21 (7)	C17—C12—C13—C14	1.54 (12)
C6—N1—C7—C8	0.24 (11)	C11—C12—C13—C14	−176.82 (8)
N5—N4—C7—N1	0.18 (11)	C12—C13—C14—C15	0.15 (13)
N5—N4—C7—C8	179.17 (7)	C13—C14—C15—C16	−1.62 (13)
C10—N3—C8—C9	0.48 (9)	C13—C14—C15—Cl1	177.25 (7)
C10—N3—C8—C7	−178.72 (8)	C14—C15—C16—C17	1.32 (14)
N1—C7—C8—N3	178.48 (7)	Cl1—C15—C16—C17	−177.53 (7)
N4—C7—C8—N3	−0.50 (12)	C15—C16—C17—C12	0.44 (13)
N1—C7—C8—C9	−0.66 (11)	C13—C12—C17—C16	−1.84 (12)
N4—C7—C8—C9	−179.64 (7)	C11—C12—C17—C16	176.54 (8)
C10—N2—C9—C8	0.18 (8)	C10—N2—C18—C19	−105.84 (9)
C18—N2—C9—C8	−175.14 (7)	C9—N2—C18—C19	68.72 (10)
C10—N2—C9—C1	−179.73 (8)	N2—C18—C19—C20	62.01 (9)
C18—N2—C9—C1	4.95 (14)	N2—C18—C19—C21	−175.26 (7)

### Hydrogen-bond geometry (Å, °)

**Table:**

*D*—H···*A*	*D*—H	H···*A*	*D*···*A*	*D*—H···*A*
N4—H1N4···O1W^i^	0.874 (19)	2.559 (18)	3.2789 (13)	140.2 (14)
O1W—H1W1···N1^ii^	0.83	2.09	2.9178 (14)	173
C10—H10A···O1W^iii^	0.93	2.52	3.3513 (16)	149
C18—H18B···O1W^iii^	0.97	2.59	3.4776 (14)	153

Symmetry codes: (i) −*x*+1, −*y*, −*z*+1; (ii) *x*+1, *y*, *z*+1; (iii) *x*, *y*, *z*−1.
